# Pseudo-Mannosylated DC-SIGN Ligands as Immunomodulants

**DOI:** 10.1038/srep35373

**Published:** 2016-10-13

**Authors:** Angela Berzi, Stefania Ordanini, Ben Joosten, Daria Trabattoni, Alessandra Cambi, Anna Bernardi, Mario Clerici

**Affiliations:** 1 Chair of Immunology, Department of Biomedical and Clinical Sciences “L. Sacco”, University of Milan, Via G.B. Grassi 74, 20157 Milan, Italy; 2 Department of Chemistry, University of Milan, Via C. Golgi 19, 20133, Milan, Italy; 3 Department of Cell Biology, Radboud Institute for Molecular Life Sciences, Radboud University Medical Center, 9101 6500 HB, Nijmegen, The Netherlands; 4 Department of Pathophysiology and Transplantation, University of Milan, Via F.lli VCervi 93, 20090 Milan, Italy; 5 Don C. Gnocchi Foundation, IRCCS, Via Capecelatro 66, 20148 Milan, Italy

## Abstract

DC-SIGN, a C-type lectin mainly expressed by DCs, mediates antigen uptake and can induce specific immune responses, depending on the ligand involved. Owing to these properties, DC-SIGN is an attracting target for approaches aimed at tailoring the immune response towards specific immunologic outcomes. A multivalent DC-SIGN ligand (Polyman26), containing at its core a fluorescent “rod-like” spacer and able to inhibit DC-SIGN mediated HIV infection in nanomolar concentration, has been recently developed by our group. We investigated the internalization pattern and the ability of Polyman26 to elicit innate immune responses. Results obtained by confocal microscopy indicate that Polyman26 is internalized by DCs via receptor- mediated endocytosis and is then routed to endolysosomal compartments, thus being presented together with MHC class II molecules, with important implications for the development of vaccines. Moreover, Polyman26 up-regulated the production of β-chemokines and pro-inflammatory cytokines (including IL-1β, IL-6, IL-12, and TNFα) as well as the expression of TLR9 and CD40L. These results indicate that glycomimetic DC-SIGN ligands should be further investigated and suggest that these compounds could be used to differentially stimulate immune responses.

Immunomodulatory strategies are currently used in the attempt to prevent and treat a range of pathologies, including viral and bacterial infection, autoimmune disorders, and cancer; these strategies, nevertheless, are still in a pioneering stage and need to be much better defined. Vaccination can be regarded as a form of immunomodulation^1^,^2^, but whether inactivated and live attenuated vaccines have intrinsic immune stimulating properties, vaccines based on recombinant antigens are scarcely immunogenic and require adjuvants to properly activate antigen specific immune responses^3^,^4^. Studies performed over the past 20 years highlighted the crucial role of the innate immune system in activating, modulating, and polarizing adaptive immunity. Results led to the suggestion that agonists of Pattern Recognition Receptors (PRRs) could be used to stimulate immune response in chronic viral and bacterial infections. Notably, some of such compounds are already in clinical use (i.e. imiquimod, as a topical treatment for Human Papilloma Virus infection) or are in advanced stages of development. In addition, PRR agonists are used as adjuvants in vaccines licensed or under clinical development^2^,^5^.

Among PRRs, C-type lectin receptors (CLRs) are promising targets for shaping immune responses^6^. Within the CLR family, DC-SIGN (Dendritic Cell-Specific Intercellular adhesion molecule-3-Grabbing Non-integrin), a receptor expressed by myeloid Dendritic Cell (DCs) and subpopulations of macrophages, recognizes specific carbohydrate structures (high mannose or fucose) on the surface of pathogens and self-glycoproteins. Upon ligand binding, DC-SIGN mediates endocytosis in endolysosomal compartments and antigen presentation *via* Major Histocompatibility Complex class I (MHC-I) and class II (MHC-II) molecules. The function of DC-SIGN can nevertheless be usurped by several pathogens, including HIV, that can exploit DC-SIGN to promote infection^7^,^8^. Indeed, DC-SIGN antagonists have been proposed for use as prophylactic antiviral agents and shown to inhibit DC-SIGN mediated infections in various *in vitro* and *ex vivo* models (for a review: ref. 9). Notably, different DC-SIGN ligands are known to induce distinct intracellular signaling pathways and immunological outcomes, supporting an important role for DC-SIGN as a target for immunomodulatory approaches^10^,^11^,^12^,^13^. Finally, thanks to its antigen uptake and signaling properties, DC-SIGN is a promising candidate for the development of vaccines targeting DCs^14^,^15^.

Synthetic carbohydrate ligands, designed to interact with DC-SIGN Carbohydrate Recognition Domain (CRD), can be used to specifically target antigens to DCs and to modulate the host’s immune response. The multivalent presentation of synthetic glycomimetic ligands on polymeric and protein scaffolds was shown to considerably increase the strength of the bond to DC-SIGN through avidity effects, and to cluster specific receptors on cell membrane, resulting in the elicitation of signal transduction pathways^16^,^17^. Similarly, multivalent dendrimers bearing glycomimetic DC-SIGN ligands performed as efficient inhibitors of DC-SIGN-mediated HIV infection of cellular and cervical explant models by competing with the ability of the virus to bind to the receptor^18^,^19^,^20^. One of the these molecules (Polyman19) could also induce the expression of β-chemokines, cytokines, and co-stimulatory molecules involved in activating DCs, in modulating adaptive immunity, and in counteracting HIV infection^21^.

Recently, we developed a novel type of glycodendrimers that include a linear rigid spacer (“rod-like” spacer) at their core^22^. This structure allows to control the size of the dendrimers and tune it to fit the distance between adjacent binding sites on a DC-SIGN tetramer, thus reaching unprecedented affinity for constructs of similar valency. Some of these molecules, appropriately loaded with DC-SIGN selective ligands, were shown to inhibit DC-SIGN-mediated HIV infection in nanomolar concentrations^23^. In the present work, compound **3.5.4** (Polyman26), the most active dendrimer in this class, was further characterized. In particular, the ability of Polyman26 to enhance and modulate innate immune responses was evaluated with the aim of developing glycomimetic ligands of DC-SIGN as immunomodulators and vaccine adjuvants. Thanks to the natural fluorescence of the rod-like spacer, the internalization pathway and the endocytic routing of the dendrimer to specific sub-cellular organelles were also investigated.

Results indicate that Polyman26 modulates multiple innate responses and is internalized by DCs into the endolysosomal compartment. These results suggest that pseudo-mannosylated DC-SIGN ligands should be further developed for immunomodulatory and vaccine approaches.

## Results

### Characterization of Fluorescence properties of Polyman26

**Figure 1:**
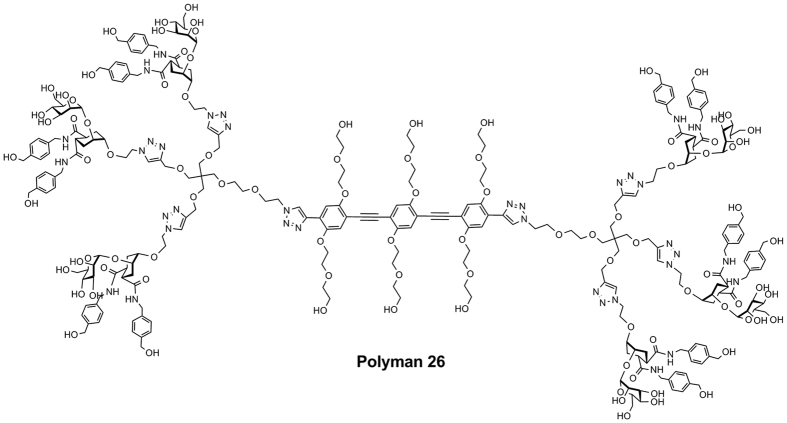
Polyman26. Structure of the polyvalent dendrimer Polyman26.

The rod-like spacer included at the core of Polyman 26 (Fig. 1) is intrinsically fluorescent; hence, it can be used as a tracer in imaging studies. To determine the optimal setting for the experiments of fluorescence and confocal microscopy, the excitation and emission spectra of Polyman26 were recorded at different pH (see Supplementary info). Results showed that irradiation at 372 nm results in a clear emission spectrum with a main peak at 425 nm and a broader shoulder at 450–460 nm (Supplementary Fig. S1), producing a readable signal also at pH compatible with acidic cellular compartments (Supplementary Fig. S2). Imaging studies were performed next to analyse the interaction of Polyman26 with different DC-SIGN domains and the ensuing internalization of the ligand.

### Binding between Polyman26 and DC-SIGN subdomains

**Figure 2:**
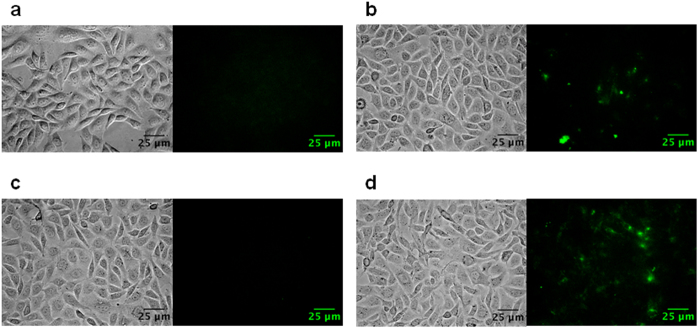
Specificity control of DC-SIGN binding to Polyman26 (pseudo-colored in green). Cells were incubated with Polyman26 for 30 min at RT; after a washing step, pictures of live cells were taken using an epifluorescence microscope. Both the bright field (left) and the signal of Polyman26 (right) are shown. **(a)** Untransfected CHO (Negative control); **(b)** CHO cells expressing DC-SIGN-wt; **(c)** CHO cells expressing DC-SIGN-ΔCRD; **(d)** CHO cells expressing DC-SIGN-ΔRep.

To determine which subdomain of DC-SIGN is targeted by Polyman26, its binding with DC-SIGN mutants lacking specific regions was evaluated. To this purpose, CHO cells expressing DC-SIGN wild type (DC-SIGN-wt), DC-SIGN lacking the CRD (carbohydrate recognition domain) (DC-SIGN-ΔCRD), and DC-SIGN lacking the tandem repeats in the extracellular neck region (DC-SIGN-ΔRep) were obtained by transfection, whose efficiency was confirmed by Flow cytometric analyses^24^. The ability of Polyman26 to bind to the cell lines harbouring the wild type or the mutated DC-SIGN was assessed by fluorescence microscopy; untrasfected CHO cells were used as a negative control. Despite the overall low fluorescence of Polyman26 stain (especially after the subtraction of the signal due to the cellular autofluorescence, induced by experimental conditions), cell images shown in Fig. 2 demonstrate that Polyman26 binds to DC-SIGN-wt and ΔRep, but not to the ΔCRD mutant. These results indicate that the lack of CRD prevents the interaction between Polyman26 and DC-SIGN, thus demonstrating that this highly mannosylated dendrimer selectively binds to the carbohydrate recognition domain of the protein (Fig. 2).

### Polyman26 internalization

**Figure 3:**
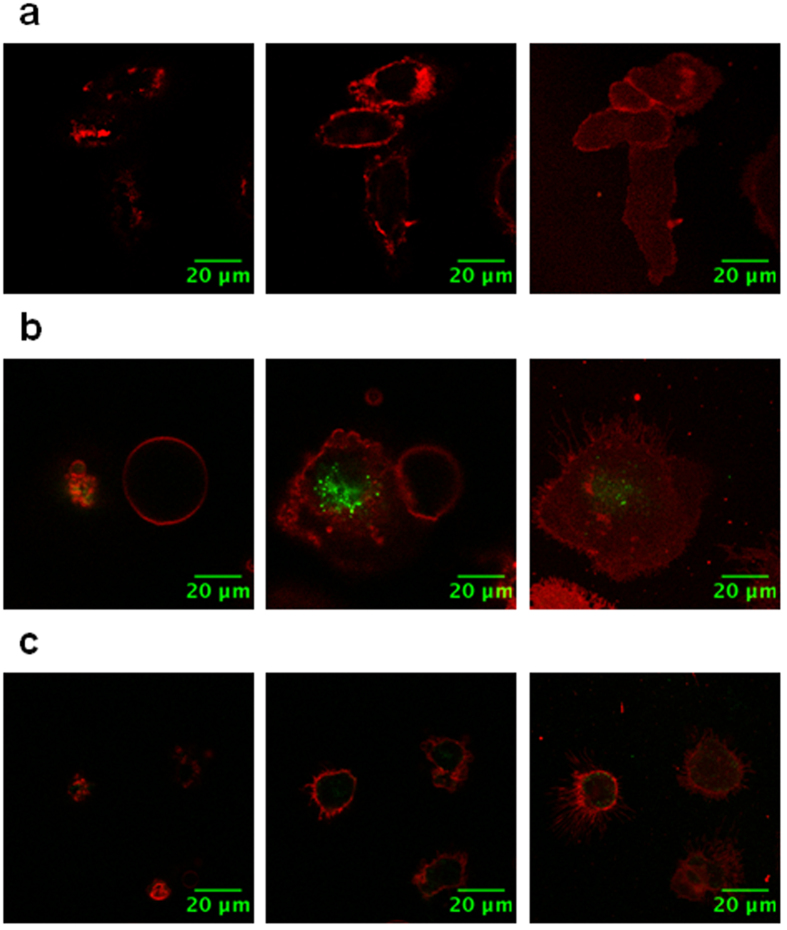
Polyman26 pulse-chase internalization experiments. Optical slices of iMDDCs incubated with 50 μM Polyman26 (pseudo-colored in green) in HBSS and, after 3 washes with HBSS (150 μL), stained for 2 min at RT with DiD’ (pseudo-colored in red) as membrane marker. After the last washing steps (2 washes with 150 μL of HBSS), cells were fixed with 1% PFA. Left: dorsal membrane (i.e. exposed to the media); centre: middle of the cell body; right: ventral membrane (i.e. in contact with the culture plate). **(a)** Negative control; **(b)** iMDDCs were incubated with 50 μM of Polyman26 at 37 °C for 60 min; **(c)** iDCs were incubated with 50 μM of Polyman26 at 4 °C for 20 min.

To study Polyman26 internalization, human immature Monocyte Derived Dendritic Cells (iMDDCs) were used as a model. iMDDCs were treated with Polyman26 for different time intervals and at different temperatures. After a washing step, *DiD’* oil (1,1′-Dioctadecyl-3,3,3′,3′-Tetramethylindodicarbocyanine Perchlorate) was used as a marker for the cellular membrane, allowing to dissect the dorsal membrane (i.e. cellular membrane exposed to the media) and the ventral one (i.e. cellular membrane in contact with the culture plate) with respect to the middle of the cell body. Pulse-chase experiments were performed, and confocal images were obtained after Polyman26 removal. For each sample, optical slices were recorded, imaging both external membrane and cross/central sections. iMDDCs with labelled membrane (pseudo-colored in red) and treated with Polyman26 (pseudo-colored in green) are presented in Supplementary Fig. S3 and in Fig. 3. Remarkably, Polyman26 was already internalized after 10 min of incubation at 37 °C (Supplementary Fig. S3a) and was still detectable inside the cells after a 60 minute incubation (Fig. 3b and Supplementary Fig. S3b, where a greater number of cells is shown). A series of optical z-sections was collected; the absence of Polyman26 on both the dorsal and the ventral membrane and its presence in the cross section confirms that the internalization of the dendrimer did occur (Fig. 3 and Supplementary Fig. S3b). Importantly, Polyman26 was not internalized when iDCs were incubated with the compound at 4 °C (Fig. 3c). Since incubation of cells at 4 °C has been described as a method to inhibit the energy-dependent internalization^25^,^26^, this result indicates that Polyman26 internalization is mediated by active mechanisms, possibly a receptor-mediated one. The lack of Polyman26 stain on cellular membrane can be ascribed to the multiple washing steps and to its intrinsic low fluorescence, which prevents observing its stain when not concentrated within cellular organelles.

### Polyman26 localization within Dendritic Cells

To study the internalization route of Polyman26 in iMDDCs and its localization in intracellular compartments, fluorescent Ovalbumin (OVA 488), Transferrin (633) and Lysotracker Red (577) were used. Transferrin^27^ is known to be internalized by cells and routed to early endosomes compartments. DCs internalize large quantities of OVA by Mannose Receptor (MR)-mediated endocytosis and small quantities by pinocytosis. While pinocytosed OVA is transported towards lysosomes, MR-endocytosed OVA colocalize with Early Endosome Antigen 1 (EEA1) positive endosomes for up to six hours after antigen uptake^28^. Therefore, once labelled with fluorescent dyes, they can be used to visualize these specific organelles. LysoTracker Red is a red-fluorescent dye for labelling and tracking acidic organelles in live cells, therefore it is selective for late endosomes and lysosomes.

Notably, antibodies could not be used to label cellular organelles because after the permeabilization procedure, required to allow antibodies to enter into the cell, and the washing steps, Polyman26 could not any longer be detected in the cells (not shown).

Confocal imaging was used to determine the degree of colocalization between Polyman26 and fluorescent tracers. After removal of unbound Polyman26, images were taken on live cells at different time intervals.

**Figure 4:**
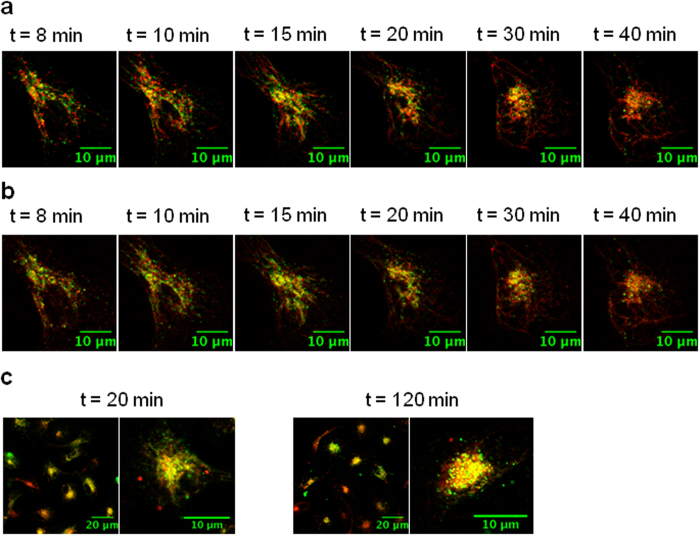
Subcellular localization of Polyman26 after internalization. Images were taken with a confocal microscope on live cells, at the time intervals indicated in the panels. Polyman26 is pseudo-colored in green and the tracers are pseudo-colored in red. **(a)** iMDDCs were incubated with Polyman26 (50 μM) plus OVA 488 in HBSS, for 10 min at RT and analysed after a washing step at indicated time points. **(b)** iMDDCs were incubated with Polyman26 (50 μM) plus Transferrin 633 in HBSS, for 10 min at RT and analysed after a washing step at indicated time points. **(c)** iMDDCs were incubated with 100 μM Polyman26 in HBSS for 20 min at RT (left) or 120 min at 37 °C (right); after a washing step, LysoTracker was added and cells were analysed.

**Figure 5:**
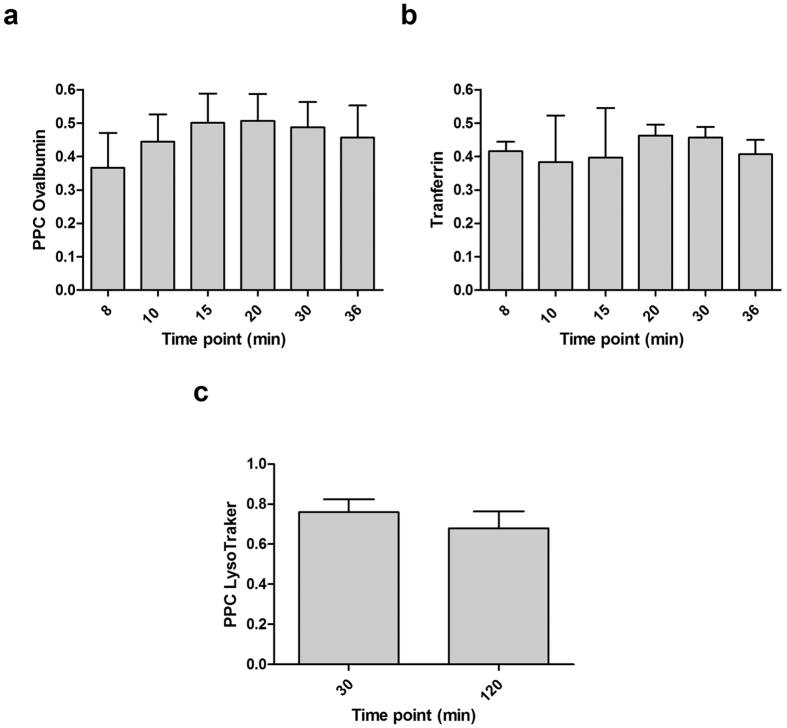
Percentage of colocalization. Pearson’s correlation coefficient (PCC) plot representing the colocalization between Polyman26 and (**a**) OVA 488, (**b**) Transferrin 633, (**c**) LysoTracker 577. Values represent the mean ± SD.

Figure 4 shows selected iMDDCs presenting Polyman26 stain (pseudo-colored in green) superimposed to the fluorescent tracer (pseudo-colored in red), for each tracer at different time points. Experiments with OVA and Transferrin were performed simultaneously, observing the two tracers at two different wavelengths on the same cells. Colocalization results in yellow spots. Both the colocalization of Polyman26 and OVA (Figs 4a and 5a) and Polyman26 and Transferrin (Figs 4b and 5b) gradually increased in time, peaking after 20 min of chase; the subsequent decrease of colocalization could indicate that Polyman26 and the tracers follow different endocytic routes (see also Supplementary Fig. S4). The highest degree of colocalization was obtained between Polyman26 and LysoTracker, reaching coefficient values >0.7 (Figs 4c and 5c). Polyman26 reached lysosomes already after 30 min of incubation, and the colocalization value was close to the one obtained after 120 min of incubation (0.8 *vs* 0.7, Fig. 5c).

### Modulation of innate immune Responses by Polyman26

Polyman26 binds DC-SIGN selectively *via* CRD; we thus evaluated the ability of the compound to activate innate immune responses. Due to the difficulty in isolating mucosal immature DCs from tissues, human iMDDCs were used in the experiments as a model^21^,^29^. Upon ligand binding, DC-SIGN activates signaling pathways that modulate Toll-like Receptor (TLR) signaling, but can also trigger innate immune responses independently of TLR activation^30^. Experiments performed showed that Polyman26 inhibit HIV *trans* infection in a clearly dose-dependent way^23^ and binds to DC-SIGN, as shown by direct interaction SPR studies with immobilized DC-SIGN Extracellular Domain (Fieschi F., Bernardi A. *et al*.; unpublished). As 10 μM was the minimum concentration of Polyman26 that completely inhibited HIV *trans* infection, we selected this concentration for functional experiments. Thus, iMDDCs were exposed to Polyman26 (10 μM) or medium culture alone (unstimulated control) in the presence/absence of Lipopolysaccharide (LPS; 1 μg/ml), an agonist of TLR4. The expression of cytokines, chemokines, and genes involved in DC activation and maturation was assessed using a Real Time PCR array specific for Dendritic and Antigen Presenting Cells. The production of cytokines and chemokines in the supernatants of iMDDCs cultures was analyzed by ELISA.

**Figure 6:**
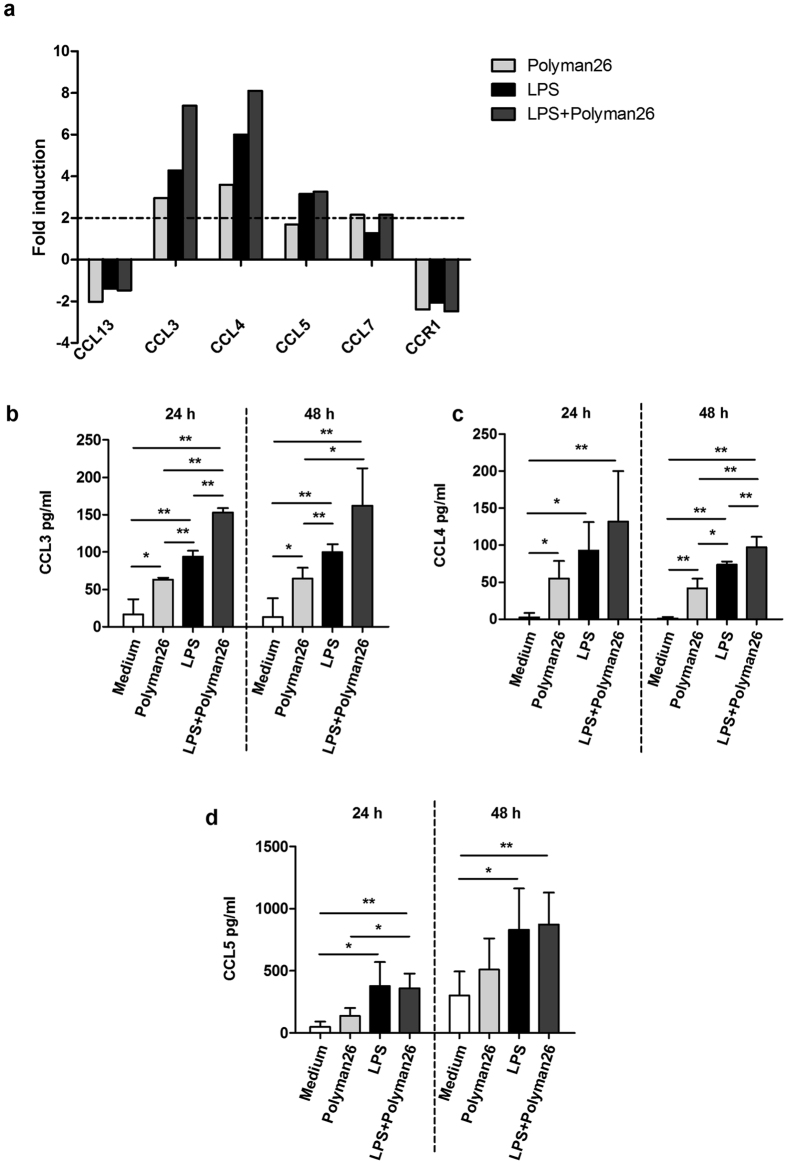
Expression of chemokine and chemokine receptors following Polyman26 stimulation. iMDDCs from 4 separate donors were treated with medium culture, Polyman26 (10 μM), LPS (1 μg/ml), or Polyman26+ LPS for 3, 24, and 48 h. **(a)** Gene expression following 3 h treatment. For each condition, cDNA was pooled and gene expression evaluated by Real-time PCR array. Expression was shown as fold induction relative to unstimulated control. Targets showing at least a 2-fold modulation were considered significant. **(b–d)** β chemokine production. The concentration of CCL3 (**b**), CCL4 (**c**), and CCL5 (**d**) in the culture supernatants was assayed by ELISA at 24 and 48 h. **(b-d)** Values represent the mean ± SD. *p < 0.05; **p < 0.01 (Student’s t-test).

Results indicated that exposure of iMMDCs to Polyman26 up-regulated the expression of the β chemokines CCL3 and CCL4 (MIP-1α and MIP-1β) compared to untreated controls; an additive effect was observed upon simultaneous treatment with Polyman26 and LPS (Fig. 6a). CCL3 and CCL4 secretion in the iMDDC supernatants was significantly up-regulated as well, both at 24 and 48 h (Fig. 6b,c). The expression of CCL7 (MCP3) was also increased by the dendrimer both in the presence and in the absence of LPS, albeit to a lesser extent. The mRNA and protein levels of CCL5 (RANTES) were significantly up-regulated upon co-administration of Polyman26 and LPS, whereas they were only modestly increased by Polyman26 alone (Fig. 6a,d). Interestingly, the expression of the HIV-1 co-receptors CCR5 and CXCR4, as well as that of DC-SIGN itself, was not modulated by Polyman26 either alone or when in association with LPS (not shown). In contrast, the dendrimer decreased the expression of the chemokine receptor CCR1 (Chemokine (C-C Motif) Receptor 1) (Fig. 6).

**Figure 7:**
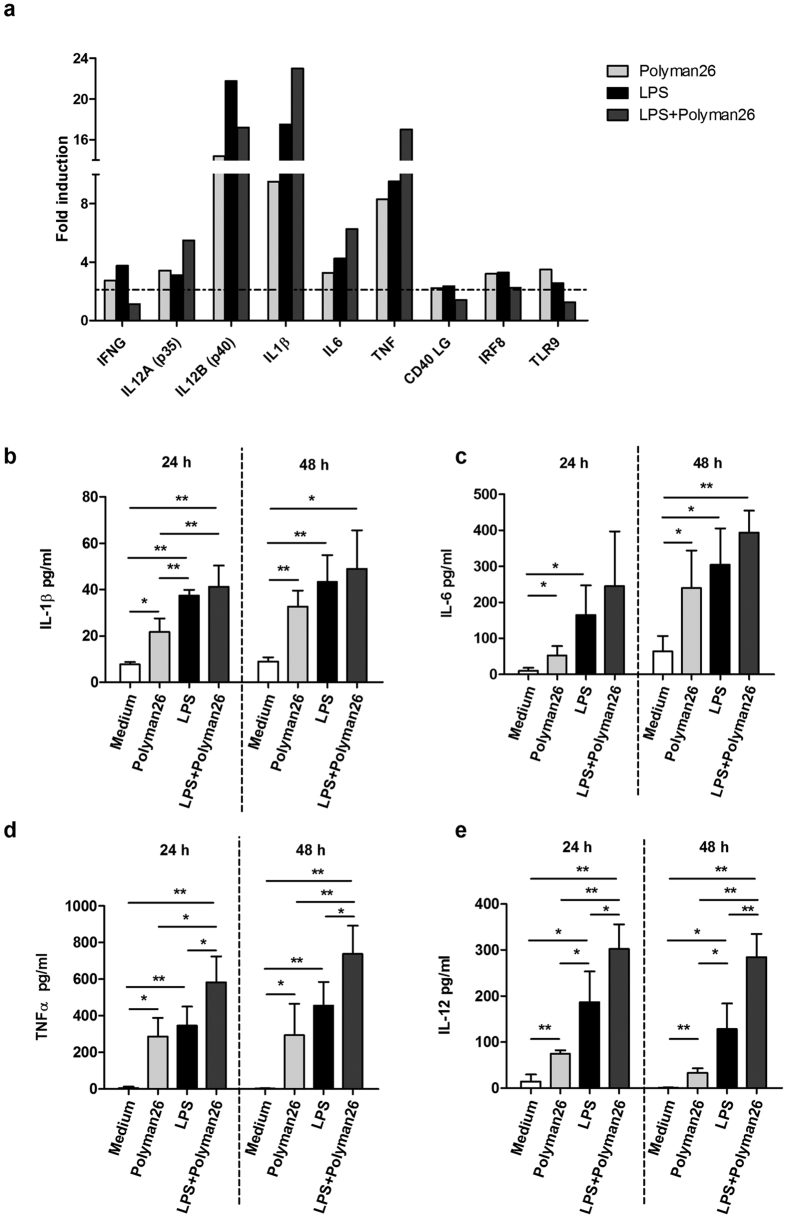
Expression of cytokines and innate immunity receptors following Polyman26 stimulation. iMDDCs from 4 separate donors were treated with medium culture, Polyman26 (10 μM), LPS (1 μg/ml), or Polyman26+ LPS for 3, 24, and 48 h. **(a)** Gene expression following 3 h treatment. For each condition, cDNA was pooled and gene expression evaluated by Real-time PCR array. Expression was shown as fold induction relative to unstimulated control. Targets showing at least a 2-fold modulation were considered significant. **(b–e)** Cytokine production. The concentration of Il-1β (**b**), IL-6 (**c**), TNFα, **(d)**, and IL-12 (**e**) in the culture supernatants was assayed by ELISA at 24 and 48 h. **(b-e)** Values represent the mean ± SD. *p < 0.05; **p < 0.01 (Student’s t-test).

Exposure to Polyman26 also resulted in the up-regulation of the pro-inflammatory cytokines IL-1β, IL-6, and TNFα. In particular, the expression and the protein production of IL-1β and TNFα were strongly increased by the compound. Also in this case, co-treatment with LPS potentiated this effect in an additive way (Fig. 7a,b,d). IL-6 was up-regulated as well upon treatment with both Polyman26 and Polyman26 plus LPS, even though to a lesser degree than IL-1β and TNFα (Fig. 7a,c).

Furthermore, Polyman26, both alone and in combination with LPS, augmented the expression of both the IL-12 subunits (p35 and p40) (Fig. 7a) and significantly increased the concentration of IL-12 in iMDDCs supernatants both at 24 and 48 h (Fig. 7e). Interestingly, an up-regulation of the transcription factor interferon regulatory factor 8 (IRF8), which is involved in the regulation of IL-12 expression, was observed as well. The compound also increased the expression levels of the Th1 cytokine IFNγ. Finally, Polyman26 induced the expression of TLR9 and CD40 Ligand (CD40 L) (Fig. 7a).

**Figure 8:**
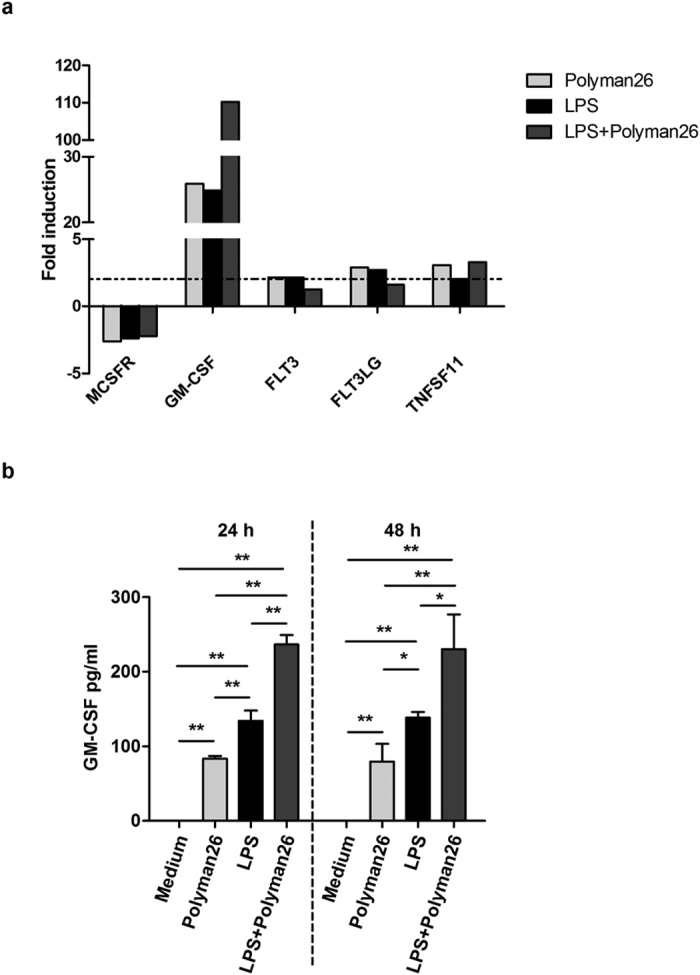
Expression of homeostatic cytokines and receptors. iMDDCs from 4 separate donors were treated with medium culture, Polyman26 (10 μM), LPS (1 μg/ml), or Polyman26+ LPS for 3, 24, and 48 h. **(a)** Gene expression following 3 h treatment. For each condition, cDNA was pooled and gene expression evaluated by Real-time PCR array. Expression was normalized to 5 housekeeping genes and shown as fold induction relative to unstimulated control. Targets showing at least a 2-fold modulation were considered significant. **(b)** The concentration of GM-CSF in the culture supernatants was assayed by ELISA at 24 and 48 h. Values represent the mean ± SD. *p < 0.05; **p < 0.01 (Student’s t-test).

Further analysis showed that Polyman26 modulated the expression of cytokine and receptors involved in DC differentiation, maturation, and homeostasis (Fig. 8). Thus, Polyman26 strongly increased the expression and the production of Granulocyte-macrophage Colony-stimulating Factor (GM-CSF), a cytokine critical for DC differentiation and maturation; also in this case, an additive effect was observed upon combined treatment with Polyman26 and LPS (Fig. 8a,b). On the contrary, Polyman26 decreased the expression of MCSFR, the receptor of Macrophage Colony-Stimulating Factor (MCSF), a cytokine which exerts an inhibitory effect on DCs differentiation. Finally, Polyman26 augmented the expression of Fms-Related Tyrosine Kinase 3 (Flt3), Flt3 ligand (Flt3L), and TNSF11, proteins that promote DC development, survival, and activation.

### Evaluation of Polyman26 toxicity

We have previously demonstrated that Polyman26 is not toxic towards a DC-SIGN-expressing cell line (B-THP1/DC-SIGN cells)^23^. To exclude potential toxic effects on iMDCCs, the cells were incubated with increasing concentrations of Polyman26, and then labelled with 7-Amino-Actinomycin D (7-AAD), which identifies non-viable cells. Exposure to the dendrimer for 3, 24, or 48 hours did not alter significantly the viability of iMDDCs up to a concentration of 100 μM (Supplementary Fig. S5).

## Discussion

Mucosal DCs, which express DC-SIGN at high levels, are among the first cell types to encounter pathogens and play a central role in the induction and modulation of the adaptive immunity. Upon ligand binding, DC-SIGN not only acts as uptake receptor but can also initiate or modulate immune responses. These features make DC-SIGN an ideal target for the development of vaccines and immunomodulants. Strategies to target DC-SIGN include carbohydrate ligands and specific monoclonal antibodies. Carbohydrate DC-SIGN ligands are less toxic and immunogenic than anti-DC-SIGN monoclonal antibodies, even though they can be less specific^15^,^31^.

We recently demonstrated that the glycomimetic compound Polyman26, a hexavalent dendrimer composed of two trivalent dendrons connected by a rod-like spacer, completely inhibits HIV-1 Bal *Trans* infection of CD4 T cells at a concentration of 10 μM and results in an inhibition of infection >98% at a concentration of 0.5 μM with an IC_50_ of 24 nM^23^.

Epithelia are effective barriers to absorption of large hydrophilic molecules. For instance, the permeability of vaginal epithelium to particles and molecules greater than 30 nm is limited^32^. However, the size of Polyman26 is certainly below that threshold. Indeed, a hydrodynamic radius of 1.3 nm was determined by Dynamic Light Scattering experiments^33^. Thus, the dendrimer could cross the epithelium, diffuse into intact mucosal tissue, and reach resident DCs. Furthermore, Polyman26 is endowed with amphiphilic properties^33^ that facilitate penetration into the subepithelial layer of the mucosa, and its absorption *in vivo* might be further improved with an adequate formulation.

The natural fluorescence of Polyman26 allowed us to study its interaction with specific DC-SIGN subdomains and to monitor its internalization pathway. Due to its suboptimal intrinsic fluorescence, Polyman26 was used at a concentration ranging from 50 to 100 μM (i.e. 5–10 times more than the concentration at which it completely inhibits HIV *trans* infection). This is a clear limitation of the system, which, however, allows to observe the behaviour of the ligand (Polyman26) in the absence of additional fluorophoric elements, which could modify its functional interaction with the cell. For instance, the label of ligands with fluorescent dyes could affect their properties, at least in terms of toxicity and binding. We therefore believe that the data, despite having been obtained with a sub-optimal chromophore, are of interest because they reflect the unbiased behaviour of the ligand in the interaction and internalization process. Results obtained by fluorescence and confocal microscopy indicate that Polyman26 specifically interacts with DC-SIGN CRD and is internalized via receptor mediated endocytosis. DC-SIGN mediates the internalization and routing of its ligands to the endolysosomal pathway, which results in peptides being presented by MHC class II molecules. Antigens targeted to DC-SIGN were nevertheless reported to be presented to CD8 T lymphocytes suggesting the existence of a still undefined mechanism of cross presentation by MHC class I molecules^14^,^15^. Our results indicate that Polyman26 ends up in lysosomes. Nevertheless, it also transits through the early endosomes, and resides there for a quite prolonged period, as shown by the colocalization with OVA and Transferrin. Since early endosomes play an important role in the cross-presentation on MHC class I, this result has important implications for the development of vaccines able to induce cytotoxic T-lymphocyte (CTL) responses.

As DC-SIGN can activate intracellular signaling with different outcomes depending on the ligand involved, we evaluated if Polyman26 could induce the activation of innate immune responses.

Results show that Polyman26, alone and in combination with LPS, induces the production of the β chemokines CCL3, CCL4, and CCL5, which are the endogenous ligands of the HIV co-receptor CCR5 and can prevent the infection by HIV-1 R5 tropic strain^34^. Thus, Polyman26, by enhancing the expression of these β chemokines, could hamper HIV infection of CCR5 expressing CD4 T lymphocytes, macrophages and DCs in genital mucosae.

Besides modulating β chemokines, Polyman26 induced an increase in the production of the proinflammatory cytokines IL-1β, IL-6, and TNFα. These results can be relevant considering that pro-inflammatory cytokines not only induce local responses that help to contain infections but also enhance the expansion and the functions of T lymphocytes. Moreover, TNFα also promotes the migration of DCs to the lymph nodes and their maturation^35^,^36^. A significant upregulation of IL-12 and IFNγ by Polyman26 was observed as well. Because both IL-12 and IFNγ skew immune responses toward the Th1 phenotype, these results suggest that Polyman26 has the ability to polarize adaptive immune responses toward such phenotype, thus promoting cell-mediated immunity against pathogens. In this context it is important to underline that local IFNγ production by DCs contributes to the early host defenses against infections and mediates autocrine and paracrine activation of Antigen Presenting Cells^37^.

Interestingly, Polyman26 also modulates receptors and cytokines involved in DC maturation, homeostasis, and activation. Indeed, Polyman26 significantly up-regulates GM-CSF, a cytokine that promotes DC differentiation and maturation. In addition, Polyman26 increases the expression of Flt3 and Flt3L, both required for the homeostatic DC development in the periphery^38^, as well as the expression of TNSF11, which acts as a DC survival factor and augments the ability of DCs to stimulate the proliferation of naïve T lymphocytes^39^. Conversely, Polyman26 reduces the expression of CCR1, a chemokine receptor down-regulated during DC maturation^40^,^41^, and the receptor of MCSF, a cytokine which impairs DC differentiation^42^. Finally, Polyman26 upregulates TLR9, and CD40L, whose expression by DCs can regulate B cell functions and the production of IgA and IgG^43^.

We previously reported that Polyman19, a glycodendrimer with a different type of core, induced the expression of chemokines and innate immunity cytokines^21^. However, a direct comparison between the effects of Polyman19 and Polyman26 on cytokine and chemokine expression cannot be drawn. Indeed, the experiments have been performed on different groups of healthy donors using different techniques and experimental settings. All these caveats notwithstanding, we observed that Polyman19 stimulation results in a more pronounced upregulation of the β Chemokines CCL3, CCL4 and CCL5, and of cytokines IL-1β, IL-6 and TNFα compared to Polyman26 stimulation. Nevertheless, it has to be noticed that an excessive production of cytokines and chemokines may cause detrimental inflammatory responses and tissue damage. Further studies in both *ex vivo* models (i.e human cervico-vaginal explants) and *in vivo* models (i.e. humanized mice or primates) will be necessary to evaluate if the levels of cytokines and chemokines induced by Polyman26 are adequate to exert immunomodulatory and adjuvant effects *in vivo*.

Cytotoxicity experiments demonstrated that Polyman26 does not exert toxic effects towards DC-SIGN expressing cell lines^23^ and iMDDCs. Nonetheless, further experiments on human tissue models (e.g. cervico-vaginal explants) should be performed to exclude potential toxic effects of Polyman26 on primary epithelial and immune cells of the mucosa and to rule out eventual damages to overall tissue integrity. Experiments on animal models will also be necessary to exclude long-term toxicity and possible toxic effects of the compound caused by the activation of the immune system.

Our findings suggest that Polyman26 may confer peculiar advantages for the prevention and the treatment of infections. As Polyman26 activates multiple effectors of the innate immunity and tailors the adaptive responses towards a Th-1 phenotype, it could offer protection against a broad range of pathogens. Furthermore, Polyman26 may potentiate the natural defenses of the host rather than targeting the pathogen, thus avoiding the development of microbial resistances. Immunomodulatory therapies could have side effects due to a generalized activation of the immune system. However, Polyman26 specifically targets DC-SIGN-expressing myeloid DCs, thus reducing the risk of side effects, especially in the case of topical administration. Further studies in the appropriate animal model (i.e. humanized mice or primates) will be necessary to validate these data and to better elucidate the effects of Polyman26 on the immune system.

Results herein show that Polyman26 is efficiently internalized by DCs via receptor-mediated endocytosis and then is routed to early endosomal and endolysosomal compartments; which has implication for the development of delivering systems that target specifically DCs and facilitate antigen presentation on MHC I and MHC II molecules. Furthermore, Polyman26 up-regulates multiple effectors of the innate immunity, including chemokines, cytokines, and receptors with important roles in the antiviral responses, in the induction of a Th1 response, and in the regulation of DC survival, maturation and activation. These results thus suggest the intriguing possibility of exploiting polyvalent carbohydrate ligands of DC-SIGN in the design of novel immunomodulants and vaccine adjuvants. Notably, as DC-SIGN is expressed by mucosal DCs, these glycomimetic compounds might specifically be used to manipulate the immune response with the aim of increasing mucosal protection against pathogens.

## Materials and Methods

### Synthesis and characterization of Polyman26

The synthesis and the characterization of Polyman26 were previously reported^23^,^33^. To exclude LPS contamination of Polyman26, Limulus Amebocyte Lysate (LAL) assay (Hycult Biotec, Netherlands) was performed. LPS content of Polyman26 at 10 μM (the concentration used to study the immunomodulatory effects of Polyman26) was below the detection limit of the assay (0.04 EU/ml).

### Cell culture

#### Transfection of CHO cells

CHO cell lines stably expressing DC-SIGN-wt, DC-SIGN-ΔCRD or DC-SIGN-ΔRep were established by Lipofectamine^TM^ 2000 (Life Technologies Invitrogen, Bleiswijk, The Netherlands) transfection^24^.

#### Differentiation of immature DCs from Monocytes

Human immature DCs were differentiated from Monocytes as previously described^19^,^21^. All experimental protocols were approved by the Institutional Review Boards of Radboud University Medical Center (Netherlands) and Vimercate Hospital (Italy). Peripheral Blood Mononuclear Cells (PBMCs) were isolated from buffy coats (Sanquin) obtained from healthy volunteers after written informed consent and all methods were carried out in accordance with the approved guidelines. Briefly, monocytes were purified from Peripheral Blood Mononuclear Cells (PBMCs) using the anti-human CD14 MicroBeads (Miltenyi Biotech, Calderara di Reno, Italy). Monocytes were differentiated into iMDDCs with hIL-4 and hGM-CSF (both 20 ng/ml; R&D Systems, Minneapolis, USA) for 6 days.

### Binding Assays between Polyman26 and DC-SIGN expressed in CHO cells

CHO cells plated on a 96-well plate were incubated with 100 μM Polyman26 in HBSS for 30 min at RT. After thorough washing with HBSS, live cells were observed using a commercial Leica DMI6000 epi fluorescence microscope with AFC equipped with a 63× oil immersion objective (HC PL APO 63x/1.40–0.60 OIL, Leica Microsystems) and filter system DAPI ET (BP 350/50, LP 400, BP 460/50), GFP ET (BP 470/40, LP 495, BP 524/50), DsRed ET (BP 546/11, LP 560, BP 605/75) and Y5 ET (BP 620/60, LP 660, BP 700/75). Polyman26 was excited at 340–380 nm. The ability of Polyman26 to bind to the different cell lines was assessed qualitatively by comparing the residual cellular fluorescence with the negative control. Images were analyzed using Fiji software.

### Polyman26 internalization in Immature Dendritic Cells

Human iMDDCs were allowed attaching to a Labtek 8-chamber slides in serum free RPMI medium. Cells were incubated with 50 μM Polyman26 in HBSS either at 37 °C for 10 or 60 min or at 4 °C for 20 min and then washed with HBSS (3 washes with 150 μL of HBSS). Cells were incubated with 100 μL of *DiD’* oil (1,1′-Dioctadecyl-3,3,3′,3′-Tetramethylindodicarbocyanine Perchlorate, Invitrogen^TM^, 9 μM) for 2 min at RT. After a washing step (2 washes with 150 μL of HBSS), cells incubated with Polyman26 at 37 °C for 10 min were observed alive. Cells incubated with Polyman26 at 37 °C for 60 min or 4 °C for 20 min were fixed with 1% paraformaldehyde for 20 min at RT, washed and blocked with 100 mM glycine in TBS (Tris-buffered saline) for 10 min at RT. Confocal images were obtained in a sequential manner using a commercial Leica SP8 SMD-WLL (Leica Microsystems) confocal microscope equipped with a PMT and Hybrid detectors (HyD) and a 63× water immersion objective (HC PL APO 63x/1.2 W motCORR CS2, Leica Microsystems). Polyman26 was excited at 405 nm. Images were analyzed using Fiji software.

### Colocalization experiments

Human iMDDCs were allowed to attach to a labtek 8 well chamber slides in serum free medium. Cells were incubated with an HBSS solution of 50 μM Polyman26 (100 μL) containing fluorescent Ovalbumin (OVA, Ovalbumin, Alexa Fluor^®^ 488 Conjugate, ThermoFisher Scientific, The Netherlands, 20 μg mL^−1^) or fluorescent Transferrin (Transferrin From Human Serum, Alexa Fluor^®^ 633 Conjugate, ThermoFisher Scientific, The Netherlands, 20 μg mL^−1^) for 10 min at RT, then a washing step was performed. In parallel, cells were incubated with an HBSS solution of 100 μM Polyman26 (100 μL) for 20 min at RT or 2 hours at 37 °C; after a washing step, 200 μL of LysoTracker Red (ThermoFisher Scientific, The Netherlands, 0.1 μM) were added. Live cells were analyzed with a commercial Leica SP8 SMD-WLL (Leica Microsystems) confocal microscope equipped with PMT and Hybrid detectors (HyD) and a 63× water immersion objective (HC PL APO 63×/1.2 W motCORR CS2, Leica Microsystems). Polyman26 was excited at 405 nm; OVA 488 at 488 nm, Transferrin 633 at 633 nm and LysoTracker Red at 577 nm. Images were taken at different time intervals to follow the intracellular localization of Polyman26 and fluorescent tracers. Images were analyzed using Fiji software. The degree of colocalization between Polyman26 and the tracers was calculated through Pearson’s correlation coefficients. The JACoP plugin of the Fiji software was used^44^.

### Microscopy equipment and settings

Images 203.44 × 151.96 micron^2^ were acquired with a resolution of 6.8421 pixels per micron (Leica DMI6000 epi fluorescence microscope, Fig. 2). Images 106.66 × 106.66 micron^2^ were acquired with a resolution of 9.6005 pixels per micron (Leica SP8 SMD-WLL confocal microscope, Figs 3 and 4). Images depth is 16 bits per pixel (Figs 2 and 4c) or 8 bits per pixel (Figs 3 and 4a,b). Magnification is 0.5.

### RNA Extraction and cDNA synthesis

RNA was extracted from iMDDCs using RNA-bee Reagent (Tel-Test Inc, Friendswood, TX, USA). To remove contaminant DNA, RNA was treated with RNase-free DNase I (New Englands Biolabs, Ipswich, MA, USA). cDNA was synthesized using Moloney murine leukemia virus (M-MLV) reverse transcriptase, in the presence of random hexamer primers, oligo DT, and RNase inhibitor (all from Promega, Milan, Italia).

### Real Time PCR

The gene expression profile upon iMDDCs stimulation with Polyman26 in the presence/absence of LPS was analyzed using the Human Dendritic and Antigen Presenting Cell RT^2^ Profiler™ PCR Array (SABioscience; Qiagen, Milan, Italy), which includes a panel of 84 genes related to DCs maturation and activation plus 5 housekeeping genes on 96-well plates. Reactions were performed using the iTaq™ Universal SYBR^®^ Green supermix on the real-time PCR detection system CFX Connect™ (Biorad, Milano, Italy). The results were analyzed exploiting the web resource GeneGlobe Data Analysis Center (Qiagen). The results were normalized to five housekeeping genes, and the relative changes in gene expression were calculated using the ΔΔCt method. Targets showing at least a 2-fold modulation were considered significant.

### ELISA

Cytokine and chemokine production in the supernatants of iMDDCs was evaluated using commercial ELISA kits following manufacturer’s instructions. The concentration of CCL3, CCL4, CCL5, IL1-β and IL-12 was measured using DuoSet kits (R&D Systems). The concentration of IL-6, TNFα, and GM-CSF was measured using Kit ELISA-Ready-Set-GO!^®^ (Affimetrix eBioscience, San Diego, CA, USA).

### Evaluation of Polyman26 toxicity

DC-SIGN expression by iMDDCs was checked by staining with the anti-human DC-SIGN PE antibody (clone AZND1, Beckman Coulter, Milano, Italy). To evaluate Polyman26 toxicity, iMMDCs were incubated with increasing concentration of Polyman26 for 3, 24, and 48 hours, and then labeled with 7-aminoactinomycin D (7-AAD) (Beckman Coulter). Samples were acquired by using a Gallios™ Flow Cytometer and data were analysed with Kaluza^®^ Flow Analysis Software (both Beckman Coulter).

### Statistic

Comparisons between the different treatments were performed using unpaired Student’s T Test for unequal variances with a two tailed P value. Significance was set at P < 0.05. Statistical analysis was performed using GraphPad Prism 5 (GraphPad Software, San Diego, CA, USA).

## Additional Information

**How to cite this article**: Berzi, A. *et al*. Pseudo-Mannosylated DC-SIGN Ligands as immunomodulants. *Sci. Rep.* **6**, 35373; doi: 10.1038/srep35373 (2016).

## Supplementary Material

Supplementary Information
