# Supplementary material for: Pseudo-Mannosylated DC-SIGN Ligands as Immunomodulants
Source: Sci Rep. 2016 Oct 13;6:35373. doi: 10.1038/srep35373 (PMC5062166; doi:10.1038/srep35373)
Supplement: Supplementary Information [file srep35373-s1.pdf]

Supplementary Information

Pseudo-Mannosylated DC-SIGN Ligands as immunomodulants

Authors: Angela Berzi[1,* +], Stefania Ordanini[2, +], Ben Joosten[3], Daria Trabattoni[1], Alessandra Cambi[3], Anna Bernardi[2], Mario Clerici[4,5]

[1]Chair of Immunology, Department of Biomedical and Clinical Sciences "L. Sacco", University of Milan, Via GB. Grassi 74, 20157 Milan, Italy

[2]Department of Chemistry, University of Milan, Via C.Golgi 19, 20133, Milan, Italy

[3]Department of Cell Biology, Radboud Institute for Molecular Life Sciences, Radboud University Medical Center, 9101 6500 HB, Nijmegen, The Netherlands

[4]Department of Pathophysiology and Transplantation, University of Milan, Via F.lli VCervi 93, 20090 Milan, Italy

[5]Don C. Gnocchi Foundation, IRCCS, Via Capecelatro 66, 20148 Milan, Italy

* corresponding author: angela.berzi@unimi.it

+ These authors contributed equally to this work

SUPPLEMENTARY INFORMATION

Supplementary Methods

Fluorescence emission measurements of Polyman26

Excitation and emission spectra of Polyman26 were recorded in a 10-mm-path length fluorescence cell, thermostated at 25.0 ± 0.1 °C, using a Perkin-Elmer fluorescence spectrophotometer. Polyman26 was dissolved in Hank's Balanced Salt Solution (HBSS) to a final concentration between 1.4 and 2.4 nM. When required, pH was adjusted to acid values by addition of hydrochloric acid, 37 wt. %.

Supplementary results

Fluorescence properties of Polyman26

Polyman26 in HBSS has an excitation maximum of 372 nm and emission maximum of 425 nm. Also a shoulder, probably corresponding to a second fluorophore, was observed in the emission spectra (Supplementary Fig. S1).

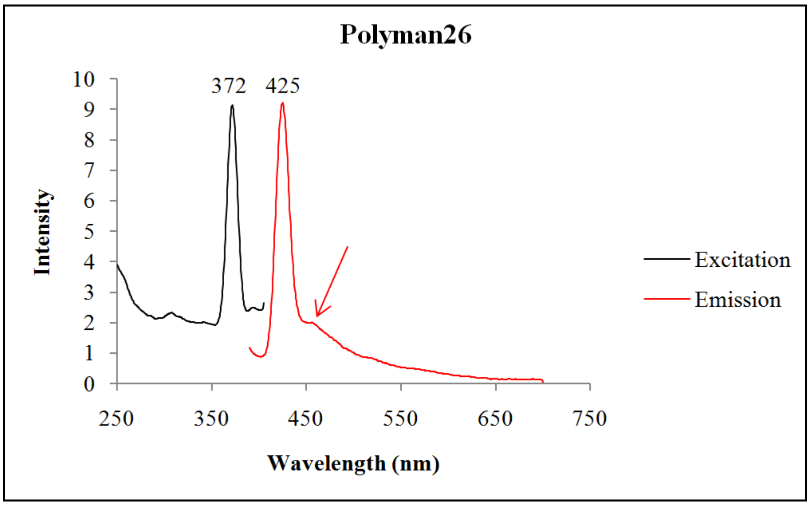

Supplementary Figure S1. Excitation and emission spectra of 2.4 nM Polyman26. The compound was dissolved in HBSS at 25 °C. An excitation peak at 372 nm can be observed. The emission profile was obtained exciting Polyman26 at 372 nm. A main peak at 425 nm and a shoulder of the main peak at 450-460 nm, indicated with an arrow, can be observed.

The emission of Polyman26 decreases by decreasing the solution pH (Supplementary Fig. S2), probably because of the protonation of the triazole moieties (pKa = 9.3) directly linked to the central rod. Remarkably, the decrease of fluorescence involves more the shoulder (450-460 nm, -54 % from pH 7.8 to pH 1.2) than the main peak (425 nm, -35 % from pH 7.8 to pH 1.2). Nevertheless, the residual fluorescence allows the compound to be detectable even once eventually internalized in acidic compartments (early endosomes pH 6.5-6.0; late endosomes and lysosomes pH 5.5-4.5).

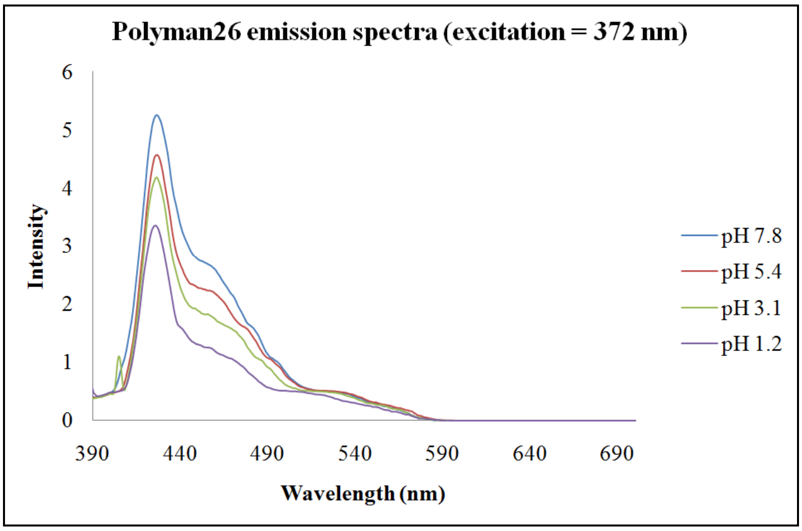

Supplementary Figure S2. Emission spectra of 1.4 nM Polyman26. Polyman26 was in HBSS at 25 °C at different pH values, as indicated in the panel. Polyman26 was excited at 372 nm.

Polyman26 internalization

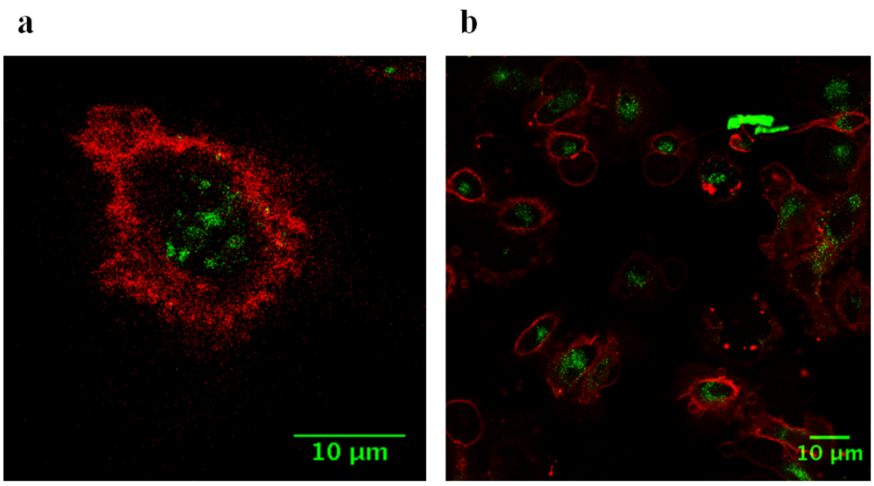

Supplementary Figure S3. Polyman26 internalization (a) Optical slices of immature Dendritic Cells incubated with 50 µM Polyman26 in HBSS (pseudo-colored in green) for 10 min at RT,

washed and finally stained for 2 min at RT with DiD' (pseudo-colored in red) to mark membranes. Samples were analysed by confocal microscopy. Polyman26 was excited at 405 nm. (b) iDCs were incubated with 50 μM of Polyman26 at 37 °C for 60 min. After 3 washes with HBSS (150 μL), cells were stained for 2 min at RT with DiD' (pseudo-colored in red) as membrane marker. After the last washing step (2 washes with 150 μL of HBSS), cells were fixed with 1 % PFA. Samples were analysed by confocal microscopy. Polyman26 was excited at 405 nm. Images 106.66 x 106.66 micron^2 were acquired with a resolution of 9.6005 pixels per micron. Images depth is 8 bits per pixel; magnification is 0.5.

Polyman26 localization within Dendritic Cells

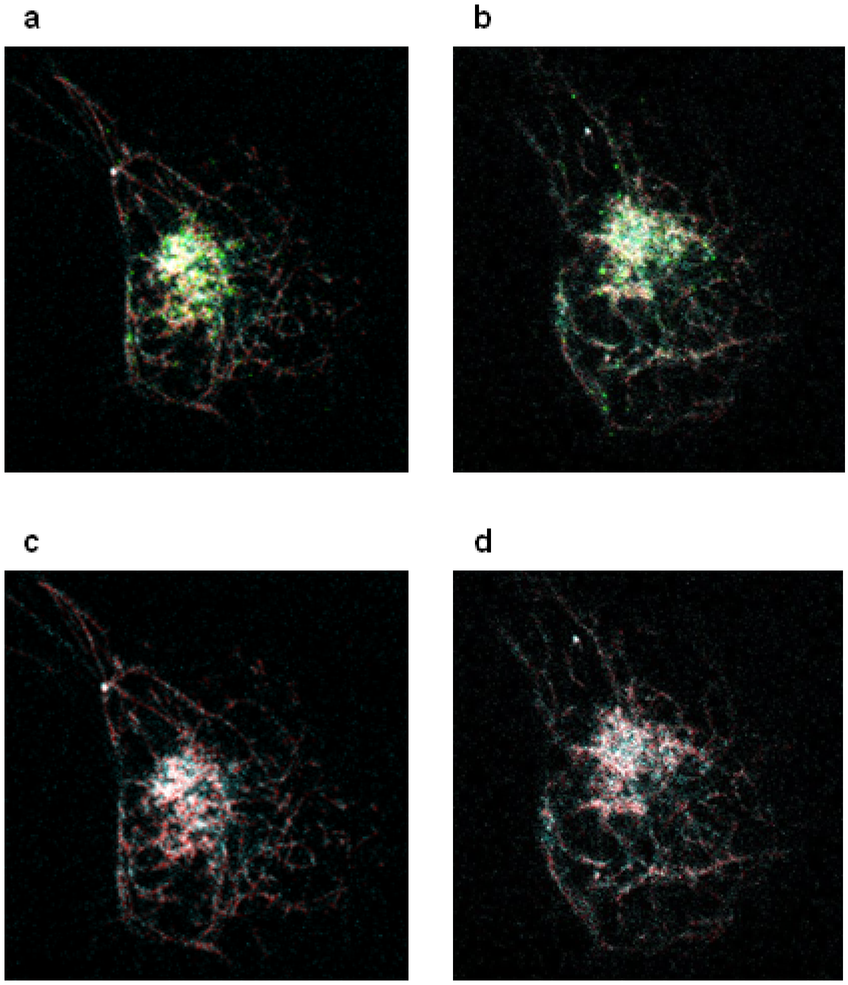

Supplementary Figure S4. Subcellular localization of Polyman26 after internalization. iMDDCs were incubated with Polyman26 (50 μM) plus OVA 488 and Transferrin 633 in HBSS, for 10 min at RT and analysed after a washing step. In the upper panel, two pictures superimposing

Polyman26 (pseudo-coloured in green), OVA (pseudo-coloured in red) and Transferrin (pseudo-coloured in cyan) at time T = 30 min (a) and T= 40 min (b). In the lower panel, OVA (pseudo-coloured in red) and Transferrin (pseudo-coloured in cyan) are superimposed at time T = 30 (c) and T=40 (d).

Evaluation of Polyman26 toxicity

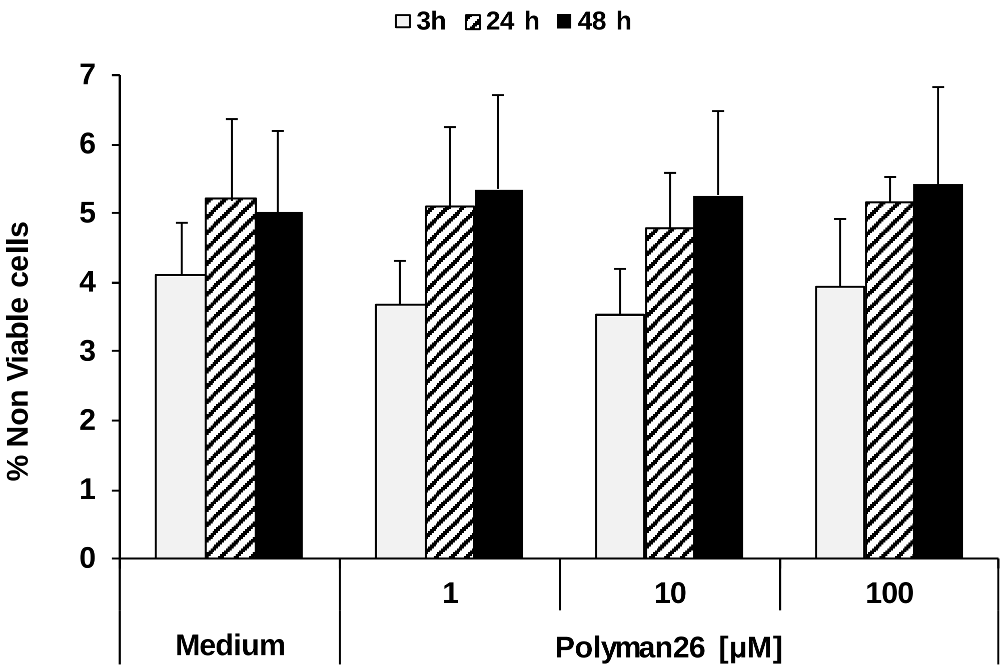

Supplementary Figure S5. **Polyman26 toxicity.** Percentage of 7-AAD positive iMDDCs (non-viable cells) after an incubation period of 3, 24 or 48h of incubation in absence (Medium) or in presence of three different concentrations of Polyman26 (1 µM, 10 µM, and 100 µM). Experiments were performed on iMDDCs from 4 separate donors. Values represent the mean ± SD.
